# Is Kraft Pulping the Future of Biorefineries? A Perspective on the Sustainability of Lignocellulosic Product Development

**DOI:** 10.3390/polym16233438

**Published:** 2024-12-07

**Authors:** Kalavathy Rajan, Paula Berton, Robin D. Rogers, Julia L. Shamshina

**Affiliations:** 1 Department of Plant and Soil Science, Fiber and Biopolymer Research Institute, Texas Tech University, Lubbock, TX 79409, USA; krajan@ttu.edu; 2 Chemical and Petroleum Engineering Department, University of Calgary, Calgary, AB T2N 1N4, Canada; paula.berton@ucalgary.ca; 3 525 Solutions, Inc., P.O. Box 2206, Tuscaloosa, AL 35403, USA; 4 Department of Chemistry and Biochemistry, Texas Tech University, Lubbock, TX 79409, USA

**Keywords:** pulp and paper, kraft pulping, environmental impact, lignin, biopolymer properties, emerging biorefinery

## Abstract

By reflecting on the history and environmental impact of conventional biorefining, such as kraft pulping, we aim to explore important questions about how natural polymers can be more sustainably sourced to develop bio-products and reduce reliance on plastics. Since the Industrial Revolution, chemical pulping processes have enabled the mass production of cellulosic products from woody biomass. Kraft pulping, which dominates within modern pulp and paper mills, has significantly contributed to environmental pollution and carbon emissions due to sulfurous byproducts and its high water and energy consumption. While chemical pulping technologies have advanced over time, with improvements aimed at enhancing sustainability and economic feasibility, conventional biorefineries still face challenges related to biomass conversion efficiency and environmental impact. For example, efforts to fully utilize wood resources, such as isolating lignin from black liquor, have made limited progress. This perspective provides a thoughtful examination of the growth of chemical pulping, particularly the kraft process, in the production of consumer goods and its environmental consequences. It also presents key insights into the bottlenecks in developing truly sustainable biomass conversion technologies and explores potential alternatives to traditional chemical pulping.

## 1. Intelligent Designs from Nature

Nature produces a diverse array of polymers, including cellulose, chitin, lignin, silk, latex, wool keratin, collagen, starch, and DNA, which can be derived from abundant and renewable sources, such as plants, animals, marine species, microorganisms, and agricultural and aquacultural waste. These natural polymers have been integral to the manufacturing of practically every product throughout human history, long before the advent of modern plastics. Examples include casein (“milk paint”), used for paint since ancient Egyptian times [1], translucent keratin slices of animal horn, used as windows dating from around the 15th century CE [2], and silk thread, which has been spun in China since the 4th century BCE [3].

Because of the diversity and complexity of natural polymers, the range of properties they exhibit is enormous and immediately apparent from their diverse natural applications where they often surpass their synthetic counterparts in many properties and functions. For instance, spider silk, made up of tightly aligned protein molecules, has a tensile strength (1.1 GPa [4]) comparable to many types of steel (1.5 GPa [5,6]), and there is speculation that reproducing dragline silk with the dimensions of 18.6 miles long and 6 mm thick would result in a strong enough material to catch a Boeing 747 mid-flight [7]. Highly crystalline chitin nanocrystals are responsible for the outstanding mechanical properties (tensile modulus 150 GPa) of the exoskeletons of crustaceans, insects, and the cell walls of fungi [8]. Chitin nanocrystals’ specific stiffness of 105 GPa (g cm^−3^)^−1^, i.e., the ratio of Young’s modulus (150 GPa) [9] to density (1.4 g cm^−3^) [10], is larger than that of metals, ceramics, or even Kevlar [11], suggesting that chitin nanocrystals could be used to fabricate lightweight and exceptionally strong materials. Alginate found in seaweed is an effective natural absorbent and accumulator of toxic pollutants [12], recommended for applications managing large amounts of exudate in wound healing, or as sorbents in environmental applications.

## 2. Woody Biomass—A Natural Smart Material

Wood, one of the most abundant natural resources on our planet, is composed of cellulose (40–60 wt.%), hemicellulose (20–40 wt.%), and lignin (10–40 wt.%) [13,14,15,16], where each of these natural polymers serves a particular purpose. Cellulose provides the wood with a tensile strength and supports plant cell walls, and its crystallinity [17,18] and chain length [19] vary depending on the species and age [20,21,22]. Hemicelluloses, heterogenous and branched polysaccharides interconnecting cellulose and lignin, also strengthen the plant’s primary walls. Lignin, with its highly amorphous and three-dimensional structure, acts as a natural adhesive, holding cellulose fibers together in plant cell walls and enhancing their rigidity [23]. Lignin gives wood its compressive strength [15], improves the plant’s hydrophobicity, and supports nutrient transport through the vascular bundles in roots and shoots [24]. Lignin also plays an important role in plant defenses against pathogens and preventing decay [25]. It is also a UV light scavenger due to its aromaticity [26].

It is reasonable to suggest that these natural polymers could be separated and repurposed to develop sustainable materials tailored to their distinct properties, addressing diverse applications. This transformative potential, first realized during the first Industrial Revolution with the emergence of the pulp and paper industry, marked a shift from an agriculture-based economy to one driven by large-scale manufacturing, paving the way for modern biorefineries. Today, lignocellulosic biorefineries represent a modern evolution of this legacy, offering a pathway toward sustainable and eco-friendly industrial practices, and further advancing the move toward a circular economy.

## 3. Kraft Pulping—How the First Industrial Revolution Caused the Emergence of a New Industry

Wood pulping, a process aimed at obtaining cellulosic fibrous material by separating cellulose fibers from wood, dates back to 1851 when the first chemical pulping process (a “soda process”) was developed and then patented by H. Burgess and C. Watt [27,28]. The process was designed to destroy the lignin and hemicellulose polymers in wood and to fully dissolve these in the pulping media, liberating purified cellulose. For this, wood was chopped, ground, and boiled in an aqueous sodium hydroxide (NaOH) solution under pressure, to create an aqueous plant fiber slurry called pulp. The pulp was then screened, bleached, shaped into sheets, and dried to make white paper. Concurrently, the process of producing a wood pulp that is almost purely composed of cellulose fibers was developed and employed the treatment of wood chips with a calcium bisulfite ((Ca(HSO_3_)_2_) [29] solution (the process is now known as sulfite pulping) [30].

**Figure 1:**
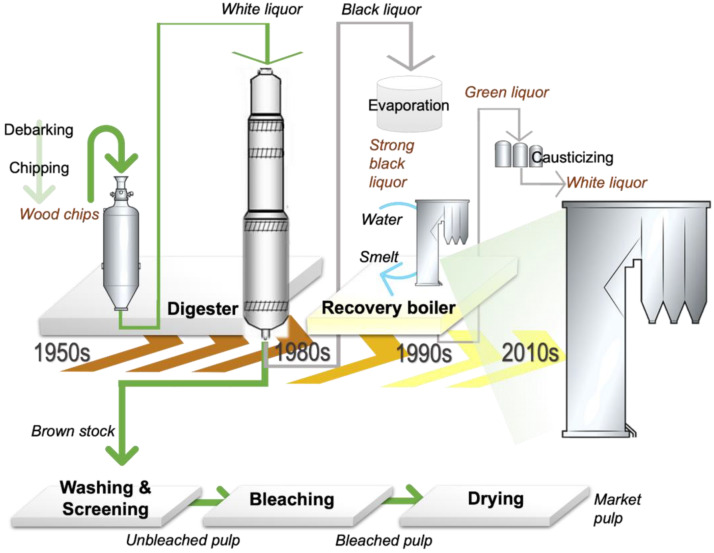
Schematic representation of a typical kraft pulping process with emphasis on the advances in woodchip digestion and black liquor treatment. Until the 1950s, woodchips were digested in a batch process, but in the 1980s continuous digesters were introduced, reducing the white liquor requirement. In the 1990s, single-drum recovery boilers were introduced. Since their introduction, these boilers have grown fourfold in capacity (as of 2016) and have significantly reduced NOx and particulate matter emissions. Adapted from [35,36].

These landmarks were soon overshadowed by an alternative method to turn wood into paper, as a more powerful separation, the kraft process (from the German “kraft”, meaning “strength”, emphasizing the production of stronger fibers), was patented in 1884 [31] (Figure 1). In this process, the wood fragments are pretreated in a mixture of aqueous NaOH and sodium sulfide (Na_2_S) at high temperatures and pressures (145–170 °C, 6–7 bar) for 2–4 h in large pressure vessels, liberating the cellulose fibers. The rest of the wood, i.e., lignin fragments (35–45%) and broken-down carbohydrates from cellulose/hemicellulose (10–15%), are released into the aqueous cooking solution which also contains sodium carbonate, sodium sulfate, and other inorganics (~15%) [32]. This by-product of kraft pulping is called “black liquor”, aptly named after its color.

A breakthrough in the kraft process was the development of the “recovery boiler” in 1933 [33] (Figure 1). The recovery boiler is where the pulping chemicals can be regenerated and subsequently reused. In this process, black liquor is concentrated down to produce “heavy black liquor” (also called “strong black liquor”) with 65–80% solids and then burned for energy generation. During combustion, Na_2_SO_4_ is reduced back to Na_2_S by the organic carbon in the mixture, and sodium hydroxide is recovered with the addition of calcium oxide. The black liquor is generally gasified and then combusted to generate electricity; a pulp mill can generate 250–500 MW of electricity via black liquor combustion [34].

There are at least 4500 active pulp and paper mills across the world [37,38,39], with the majority located in Asia (~3200) [39,40]. These pulp and paper mills produce approximately 165 million tons of cellulose pulp and 145 million tons of paper and paperboard. Of these, 128 paper mills operate in the USA [41], generating 46.6 million tons of cellulose pulp [42], with the kraft process accounting for over 80% of pulp production [43]. On the other hand, the demand for paper products is rising at a projected rate of 6.1%, from 414 million tons in 2022 to 476 million tons in 2032 [44]. Since 80% of the global pulp is produced via chemical refining [45], such as kraft pulping, where every ton of pulp generates about ~10 tons of black liquor (~10% dry matter) as waste, it is essential to understand the environmental and economic impact of such conventional processes [46,47].

## 4. Ongoing Environmental and Economic Sustainability Issues

### 4.1. Operational Challenges

Pulp mills are reported to have a higher incidence of both fatal and non-fatal accidents (18 to 28 per 1000 employees) compared to sectors like petroleum and coal manufacturing, which report 15 accidents per 1000 employees [37,48]. Workers in pulp mills face health risks due to the high temperatures and the handling of chemicals and materials [49]. Additionally, the mills encounter ongoing operational challenges with equipment such as multi-effect evaporators, recovery boilers, lime kilns, and causticizing plants, which can lead to issues like corrosion, cracking, smelt-water explosions [50,51], unsteady smelt run-off [52], increased energy consumption, and higher air emissions [53,54]. The chemical recovery process and reduction in toxicants from various aqueous effluents of kraft pulp mills—including steam condensate, bleaching, non-process elements, and waste disposal systems—also present significant challenges, particularly due to blockages and hazardous leaks [55]. These operational difficulties often lead to increased costs, which may deter some countries, particularly low-income nations, or those with limited regulations, from adopting safer and more environmentally friendly chemical pulping practices.

### 4.2. Environmental Problems

**Hazardous Aqueous Effluents:** On average, kraft cooking, washing, and bleaching processes, require 45,000 L of water per ton of dry pulp [56]. At 1.5 trillion gallons per year, pulp mills are the largest consumer of processed water in the USA, discharging 330 billion gallons [57] of scrubbed wastewater. The untreated aqueous effluents from chemical pulping may contain about 250 different compounds (chlorinated organic compounds, dioxins, chlorobenzenes, metalloids, fatty and resin acids, sulfuric acid, Hg, N, P, Ca, Mg, trace elements, etc.) [58,59,60], which could prove to be significantly toxic to aquatic life even at a 2% concentration [61]. A key indicator of water pollution from pulp mill effluents is the biochemical oxygen demand (BOD), which measures the oxygen needed by microorganisms to break down organic pollutants [62]. High BOD levels can deplete oxygen in water bodies, threatening aquatic life and disrupting aquatic ecosystems. Federal organizations such as the U.S. Environmental Protection Agency [63,64], European Integrated Pollution Prevention and Control [65,66], and the Canadian Environmental Protection and Conservation Department [67], have implemented many control measures (in the late 1990s and early 2000s) to prevent toxic levels of contaminants from entering their water resources. They monitor about 21 different metrics to assess the environmental toxicity of pulp mill effluents. To reduce the discharge of noxious pollutants, good manufacturing practices such as the imposition of fines and closed-loop water circulation systems are strongly recommended. Although pulp mills in the USA, Canada, and Europe often treat their aqueous effluents effectively, they do not entirely eliminate the hazardous pollutants, leaving behind a toxic legacy for future generations [68,69,70].

**Air Emissions:** One of the most significant types of emissions from kraft mills are sulfurous compounds containing sulfur in its reduced state, collectively referred to as Total Reducing Sulfur (TRS). For every ton of dry pulp, kraft mills produce up to 3 kg of sulfur-containing gasses such as hydrogen sulfide (H_2_S), methyl mercaptan (CH₃SH), dimethyl sulfide (DMS), and dimethyl disulfide (DMDS) and 30 kg of sulfur oxides (SO_x_) [71,72]. These compounds are released during the chemical pulping process, where wood is treated with a mixture of NaOH and Na_2_S. In addition to sulfur compounds, 15 kg of other volatile organic compounds (VOCs, e.g., benzene, *p*-cymene, toluene, chloroform and terpenes) are produced by kraft mills [71,72]. Turpentine oil, composed of terpene hydrocarbons (including *α*-pinene, *β*-pinene, limonene, 3-carene, and camphene), is emitted in amounts up to 6 kg [71,72], primarily from the wood itself during the pulping process. These compounds contribute to the characteristic odor of kraft mills and can also contribute to the formation of ground-level ozone, which is a key component of smog. While not as immediately harmful as some other pollutants, VOCs like terpenes can still have a negative impact on air quality and contribute to broader environmental issues. The sulfur compounds also contribute to the characteristic odor associated with kraft mills and can have detrimental environmental and health effects. Kraft mills also emit 150 kg of fine particulate matter (PM), which includes PM2.5 (particles with a diameter of 2.5 µm or smaller) and PM10 (particles with a diameter of 10 µm or smaller). These particles can originate from the combustion processes in the recovery boiler and other mill operations. PM2.5 is especially concerning as it can penetrate deep into the lungs, leading to respiratory problems and cardiovascular diseases. PM10 is larger but still poses health risks, particularly for workers in close proximity to emission sources. Even though the industry cut their emissions by ~85% from 1973 to 1990 [73], significant challenges persist. These include the formation of sulfur dioxide (SO_2_) during the combustion of black liquor in recovery boilers and lime kiln operations, contributing to acid rain and respiratory issues. The distinct odors from sulfur compounds, the role of VOCs in forming ground-level ozone, and the need to comply with stringent environmental regulations (e.g., U.S. EPA’s Clean Air Act standards and the European Union’s Integrated Pollution Prevention and Control Directive) further complicate air emissions management. Addressing these challenges often requires advanced and costly air-scrubbing and filtration technologies.

**Other Environmental Problems:** The pulp and paper industry is also among the top five most energy-intensive industries globally and is the fourth largest industrial energy user [74]. It accounts for approximately 6% of global industrial energy use, 1.3% of global greenhouse gas (GHG) emissions (or ~2% across the whole process lifecycle) [75], and 2% of direct industrial carbon dioxide (CO_2_) emissions. Of the 1.3 million tons of CO_2_ emitted by a typical pulp and paper mill in the U.S., every year, more than 65% stem from the use of a recovery boiler. Even though CO_2_ is derived from the burning of natural sources, it still contributes to the overall emissions of the production process. Worldwide, the pulp and paper industry generated 43.5 billion tons of CO_2_—eq GHG emissions (standardized to that of one-unit mass of CO_2_, based on the global warming potential (GWP) of the gas), accounting for 4.2% of global anthropogenic GHG emissions between 1961 and 2019 [76]. Thus, the benefits of kraft pulping are severely undermined because of the long-term adverse environmental impacts.

**Regulations:** Kraft pulping is heavily regulated globally due to its potential environmental impacts, particularly regarding air and water emissions. In the United States, the Environmental Protection Agency (EPA) subjects kraft pulp mills to a variety of different key regulations such as the Clean Air Act (CAA) and Clean Water Act (CWA) [77]. Under the CAA, the EPA targets pollutants like SO_2_, TRS compounds, and PM, requiring mills to adopt advanced technologies such as scrubbers, electrostatic precipitators, and low-NOx burners. The CWA, on the other hand, regulates wastewater discharge, mandating effluent treatment to minimize harmful substances such as BOD, chlorinated organic compounds, and heavy metals. The Cluster Rule, introduced in the 1990s, specifically targets the kraft pulping process within the pulp and paper industry, aiming to reduce toxic air emissions and wastewater pollutants from these facilities by combining air and water quality standards under a single rule [78].

In the European Union, the Integrated Pollution Prevention and Control (IPPC) Directive, now part of the Industrial Emissions Directive (IED), mandates that pulp mills adopt Best Available Techniques (BATs) to prevent and minimize emissions to air, water, and land, essentially forcing them to prioritize pollution prevention at the source rather than just treating it afterwards [79]. These techniques include reducing TRS emissions, lowering the chemical oxygen demand (COD) in wastewater, and implementing energy-efficient processes. Similarly, in Canada, the Canadian Environmental Protection Act (CEPA) focuses on minimizing the release of toxins such as dioxins, furans, and VOCs, while encouraging closed-loop systems and renewable energy use, including those associated with kraft pulp and paper processes [80]. Other countries, like India [81] and China [82], have their own environmental regulations targeting air and water pollutants from kraft mills. By pushing for compliance with stringent standards, these regulations encourage a shift toward renewable energy and circular economy principles, aligning Kraft pulping with modern sustainability goals.

### 4.3. Low-Quality Co-Products

Modern pulp mills function similarly to biorefineries, aiming to maximize the utilization of all biopolymers and depolymerized waste streams [83]. For example, Borregaard (Sarpsborg, Norway), one of the world’s most advanced biorefineries in Norway, produces ethanol, lignin, vanillin, and biogas, alongside conventional cellulose pulp. [84]. However, many pulp mills worldwide face challenges in isolating and valorizing their co-products. This difficulty arises because the high temperatures and pressures used in chemical pulping degrade the molecular weight (MW) of the wood polymers, which is crucial for determining their potential applications [85,86]. Kraft pulping is not only inefficient in complete lignin removal but also degrades 75% [87] of the hemicelluloses because of both high temperatures and strong alkaline conditions [87,88,89]. Purification of the hemicelluloses from black liquor is also challenging because of ash and lignin contamination [90]. This could be considered a significant loss since hemicelluloses form 20–40% of wood and could be used to produce specialty chemicals (e.g., xylitol) [91], platform chemicals (e.g., furfural) [92], adhesives [93], biofuels [94], etc.

As for lignin, it mostly degrades to fragments that are soluble in a strongly basic solution. When the chemical properties of kraft lignin from mixed hardwoods (*Acacia* and *Quercus* spp.) were compared to that of ball-milling (using standard toluene extraction [95]), the M_W_ of milled wood lignin was in the range of 12,000–13,000 Da whereas the M_W_ of kraft lignin was ~3000 Da (Table 1). Thus, kraft pulping produces a polymer with severe degradation [96]. This is in line with observations obtained by others for the kraft pulping of loblolly pine, 3350–4400 Da [97]. Such a loss limits the possible applications of lignin and reduces its marketability. The opportunity cost of this loss is substantial. According to industry estimates, the chemical pulping industry could lose between USD 900 million and USD 1.6 billion in potential revenue from the underutilization of these co-products [98,99]. If these valuable polymers could be preserved and properly isolated, they could be used in a variety of high-value applications. The market impact of losing these co-products not only deprives the industry of potential revenue but also reduces the economic viability of pulp mills, especially as the demand for bio-based and sustainable products continues to rise. To unlock this potential, it is crucial to develop more efficient technologies that can prevent polymer degradation during the pulping process and allow for the extraction and valorization of these co-products [100].

Extensive research has been conducted to valorize such low-quality lignin co-products, which, due to the depolymerization during kraft pulping, result in lignin with a significantly reduced MW. We would like to emphasize here that these **low-quality lignins are not the optimal products** that could be obtained from higher MW lignin, which would be more suitable for high-value applications. Despite their reduced quality, these depolymerized lignins still possess properties that make them candidates for a range of bio-based materials. As will be discussed in Section 5.2, ongoing efforts are exploring ways to effectively utilize these low-quality lignins in the preparation of various biomaterials, aiming to unlock new opportunities for sustainable production and further reduce reliance on fossil-based resources.

## 5. The Industry’s Efforts to Sustain Convention and the Bottom Line

Global socioeconomic and political changes have affected the chemical pulping industry’s supply chain in recent decades [103]. A shrinking bottom line due to an increase in the price of the raw materials accounts for 30–40%, sometimes >50%, of the total product cost. Second, the paper and pulp industry are being stressed to shift towards a more sustainable future and to implement eco-friendly paper manufacturing practices, lessen/offset their carbon footprint, and reduce waste. This necessitates significant changes in existing technological processes, upgrades to the manufacturing equipment that affect capital costs (CAPEX), improvements in process efficiency, and reductions in water and energy usage. Stringent federal and state regulations related to emissions, including persistent organic pollutants, total suspended solids, and GHG, impose additional costs on paper mills. In addition, there are costs associated with chemical waste management, disposal, and water usage. As a result, the pulp and paper industry face new challenges from decreased profitability today, requiring significant investments to sustain its overall financial health.

To address these challenges, paper mills have been implementing several tactical approaches to revitalize the paper sector. Black liquor contains a significant amount of organic material, primarily lignin, which has a high calorific value. With an energy content of approximately 14 MJ/kg of dry solids—roughly half the energy content of 1 kg of a coal equivalent—it serves as an efficient fuel source. The steam and electricity produced from burning black liquor not only power the mill’s operations but also, in some cases, generate surplus energy that can be sold to the local power grid [104]. Radiant-type recovery boilers that were introduced in the 1980s feature an improved design that enhanced heat transfer, resulting in efficient high-pressure steam generation compared to the previously used bi-drum boilers. The steam powers turbines, producing electricity and mechanical energy for mill operations. This innovation improved mill self-sufficiency, reduced dependence on external energy sources, and increased the capacity to sell surplus energy. Today, pulp mills use advanced radiant recovery boilers with more efficient air distribution, integrated sensing, and NO_x_-reducing catalytic converters [105,106,107]. Their black liquor combustion capacity has expanded to 12,000 dry tons/day, enabling pulp mills to generate up to 12,000 MWh of excess energy [108,109].

While modern radiant boilers are efficient, the primary focus remains on improving energy recovery rather than introducing fundamentally new capabilities. This approach makes existing processes more cost-effective but does not fundamentally alter how black liquor is utilized or expand the range of outputs it can generate. Enhancements in energy output, emission reductions, and chemical recovery represent incremental rather than transformational improvements.

Instead of burning black liquor solely for energy, it can be repurposed for higher-value applications through innovative technologies. Advances in research have enabled the emergence of systems like the black liquor gasification (BLG) system, where the recovery boiler is replaced with a gasification plant. BLG involves exposing black liquor to high temperatures in the presence of controlled amounts of oxygen or steam-producing syngas, a mixture of hydrogen (H_2_) and carbon monoxide (CO) [34,110]. Syngas offers greater flexibility compared to direct combustion, as it can be used not only as a fuel but also as a raw material for synthesizing higher-value products. This approach diversifies revenue streams by enabling the production of valuable platform chemicals (phenolics, cyclopentenone) from black liquor which may generate new, higher sources of income, rather than limiting outputs to energy alone. Pioneering companies such as Chemrec AB (Piteå, Sweden) and Georgia-Pacific (Emporia, VA, USA), began adopting black liquor gasification systems in the early 2000s. BLGs can increase the amount of electricity generated at the pulp mill by two to three times. Moreover, new supercritical water-based gasification technologies are being developed to produce hydrogen and valuable platform chemicals (phenolics, cyclopentenone) from black liquor, which may generate new, higher sources of income [111,112]. However, the investment required for a full-scale BLG process unit is larger than that for a new conventional recovery boiler, and commercial BLG systems are still relatively small. Thus, Weyerhaeuser (Elkin, NC, USA) processes 330 tons of black liquor solids per day, Georgia-Pacific (VA, USA) processes 200 tons of black liquor solids per day, and Norampac (Vaughan, ON, Canada) processes 100 tons of black liquor solids per day [113]. Other ways of utilizing black liquor are lignin precipitation from black liquor by acidification (the LignoBoost process [114], discussed below) and the upgrading of tall oils to produce biodiesel [115].

In addition, the industry has been improving lignin isolation so that it could be sold as a value-added platform chemical. Borregaard LignoTech (Sarpsborg, Norway), for example, has production facilities in six different countries [116] and sells ~600 different lignosulfonate products to more than 3000 customers around the world [117]. The Ingevity plant in Charleston, SC produces ~40,000 metric tons of the polymer yearly. Stora Enso’s plant (Helsinki, Finland) has an annual capacity of ~50,000 metric tons of lignin [118], whereas UPM Biochemicals [119] and FPInnovations (Pointe-Claire, QC, Canada) [120] produce 20,000 and 10,500 metric tons of lignosulfonates, respectively. Advanced processes like LignoBoost (Valmet Power AB, Aiken, SC, USA) [121] and LignoForce (FPInnovations, Pointe-Claire, QC, Canada) [122] were patented in 2006 and 2011, respectively, to isolate kraft lignin via two-stage acidification or sequential oxygenation and carbonation. Both processes improved the functional properties of kraft lignin and reduced hazardous (sulfur-containing) impurities. New methods are also being tested to improve the homogeneity and commercial value of lignin, e.g., the sequential solvent fractionation of kraft lignin, enzymatic polymerization and depolymerization, and nanoparticle synthesis [123,124,125]. The second tactic is focused on allocating resources towards research and development (R&D) to add new products to the pulp and paper mill portfolio and penetrate other profitable markets; we will discuss these strategies in the following subsections.

### 5.1. “Drop-In” Chemicals to Shift Towards “Bio-Based” Solutions

Pulped cellulose and lignin can be used as a replacement for petroleum-based chemicals, such as ethylene derived from bioethanol to make bio-polyethylene [126]. “Drop-in” raw materials are structurally identical to their fossil fuel-derived peers but instead are produced from wood. Lignin, exploited for its aromatic backbone, can be dropped into the manufacturing of different consumer products, such as adhesive resins [127], activated carbon and carbon fibers or mats [128], hydrogels [129], microspheres [130], membranes [131], 3D constructs [132], etc. Moreover, lignin, along with cellulose and hemicellulose, can be hydrolyzed into its monomeric form and then upgraded into platform chemicals like furfural, organic acids, monophenols, and polyols that serve as precursors to bioplastics, bioactives, and hydrocarbon fuels [133]. Lignin can also be converted into carbon products like carbon foams, battery anodes, and carbon fibers [134].

As the name implies, “drop-ins” already have a current market demand and can essentially be dropped into existing manufacturing processes without changing the main plant operations. Challenges for “drop-ins” include non-competitive prices, but there are no requirements for CAPEX investments associated with obtaining the raw polymers, as these come from kraft pulping. While there is a difference in terms of the raw material (fossil fuel versus wood), there is unfortunately no difference between “bio-based” drop-ins and petrochemicals when looking at the end-of-life solutions.

### 5.2. Greenwashing

The practice of companies’ expansion into new products and/or markets to reduce their financial risks may lead to “greenwashing”, where non-sustainable technologies benefit over genuinely sustainable ones. The pulp and paper industry, for instance, is well-positioned to capture the growing market for biodegradable packaging alternatives, which is projected to expand at a compound annual growth rate (CAGR) of 10% from 2023 to 2032 [135]. Valued between 19 billion and 810 billion USD, the shift toward paper-based products in global packaging is seen as a potential solution to the environmental crises caused by plastic. However, the full costs of this transition remain uncertain. The European Paper Packaging Alliance, for example, advocated for single-use paper packaging over multi-use products in the name of reducing CO_2_ emissions, fine particulate matter pollution, and metal depletion [136]. However, they failed to account for the carbon footprint of deforestation or the carbon sequestration potential of multi-use paper products. While the recycling of paper products (e.g., recycled paper-based packaging, bioethanol from cardboard) [137] has been universally accepted as being eco-friendlier than incineration, landfilling, or continued production of virgin wood fiber [138], such technologies must be comprehensively evaluated for energy, water, and chemical use to avoid unforeseen environmental damage. Similarly, a review of the Life Cycle Analyses (LCAs) that summarized 42 environmental LCA studies of lignin-based products [139] revealed that while many of them considered the end-of-life scenarios, the impact categories that dealt with resource use (e.g., land and water), emissions during the pulp production, and the overall climate impact were often overlooked.

We would like to advocate here for a push towards sustainability that will translate into the development of totally different technologies than those we currently utilize, technologies that are not contaminating our soil and groundwater, and whose GHG emissions are negligible while producing high-quality polymers. While we could make cheaper materials from toxic waste to increase an industry’s profits, this will not bring us closer to an eco-safe future. If we are not careful, the impulse to “greenwash” bio-based but environmentally detrimental products will suppress the development of genuinely sustainable processes.

## 6. A Responsible Future for the Pulp and Paper Industry

To be profitable, the global pulp and paper industries necessitate gigantic economies of scale and a constant reduction in operating costs. As McNutt claimed in his article, a mature, capital-intensive, extremely cyclical industry is “seriously affected by failing performance and returns, monolithic and slow to change. Substantive assets are underutilized and under-performing. Leadership seems largely to lack adequate vision, innovative thinking, and a good solid understanding of the character of value” [103]. To address these challenges and achieve sustainability in the pulp and paper industry, potential solutions include adopting environmentally friendly processes such as IL- or DES-based fractionation to reduce the chemical impact and improve efficiency. Maximizing the recovery and utilization of lignin and hemicellulose with higher MWs for bioplastics, while repurposing degraded carbohydrates for specialty chemicals, can enhance co-product value. Additionally, implementing closed-loop systems to recycle waste and reduce resource consumption, utilizing black liquor gasification or combined heat and power (CHP) systems to improve energy efficiency, and exploring biorefinery models to diversify product portfolios and generate higher-value products are essential steps. These transformative measures represent a shift from minor iterative changes to a more innovative and sustainable approach.

### 6.1. Artificial Intelligence: The Answer to Every Chronic Problem

A “bottom-up” measure to address the environmental and economic sustainability issues of the pulp and paper mills would be digital reformation. The large quantities of existing production and consumption data can be leveraged to unlock connectivity across the value chain, i.e., consumer demand, forest resources, water- and energy-use management, milling, and downstream chemical recovery [140]. Advanced data analytics could enhance machine connectivity and intelligent automation yielding 5 to 10% input savings as well as high throughput gains for the pulp mills [141]. For example, machine learning can reduce downtime, energy usage, resource inputs, product defects, and waste, with predictive maintenance saving up to USD 500,000 per machine annually. Tools like eLIXA^®^ by Haber Inc. (Pune, India) dynamically optimize chemical inputs by measuring key parameters in the pulping liquor process in real-time, while platforms like PULMAC (Williston, VT, USA) blend human expertise with analytics, and ProcessMiner™ (Atlanta, GA, USA) aims for complete automation. The next generation of AI platforms is poised to integrate neural networks and deep learning, using data envelopment analysis to enhance paper yield and minimize environmental impacts [140,142]. By harnessing the full potential of AI, the pulp and paper industry can achieve a paradigm shift toward unprecedented efficiency, sustainability, and innovation.

### 6.2. Sustainable Feedstock Supply

In recent years, top pulp manufacturers and tree farms have been required to follow sustainable forest management practices through reforestation, reducing waste, maximizing efficiency, and through certifications from environmental management systems like the Forest Stewardship Council (FSC), Sustainable Forestry Initiative (SFI), and the Program for the Endorsement of Forest Certification (PEFC) [143]. Concerns about deforestation and the opportunity for shorter carbon cycling have led to the boom of the ‘tree-free fiber’ movement, where crop residues like sugarcane bagasse, dedicated energy crops like *Miscanthus*, and traditional fiber crops like bamboo, are used as alternatives to wood for pulp manufacturing [144]. Additionally, recovered paper and cardboard have become increasingly important as sustainable feedstocks in pulp production, contributing to the circular economy by reducing the demand for virgin fibers [145,146]. Recycled paper products, such as corrugated containers and newsprint, which totaled 46 million tons of paper at a recycling rate of 65–69% and cardboard at a recycling rate of 71–76%, play a significant role in the cascaded production of cellulose, cellulose nanocrystals, and hydrocarbons [147,148]. The next generation of biorefineries must accommodate a variety of feedstocks, including woody, herbaceous, and recycled fibers, to ensure sustainable and efficient operations. Companies that invested in paper recycling capacity increases since 2017 include Packaging Corp. of America, Copamex, Pratt Industries, Bio Pappel, International Paper, Nine Dragons, Graphic Packaging, Domtar, Sonoco, and Cascades, among many others.

At the same time, recycled paper faces several challenges, including the degradation of fiber quality with each recycling cycle, which reduces the fibers’ strength and suitability for high-quality products. Contamination with inks, adhesives, and non-paper materials requires additional processing and increases costs. Mixed paper grades and non-paper items complicate sorting and recycling, further lowering the quality of the pulp. Certain paper types, like coated or laminated papers, are difficult to recycle, while others, such as heavily inked papers, require extensive deinking processes. Additionally, the supply of high-quality recycled paper may be inconsistent, and the market demand for recycled products can be limited. Finally, the deinking process involves chemicals and water, which can have environmental impacts. A balanced mix of both “virgin” and recycled fibers would ensure high-quality products while promoting sustainable forest management.

### 6.3. Disruptive Innovations in Wood Pulping

In the design, research, and decision-making processes for the expansion of the use of drop-in raw materials in pulp mills, the life cycle, health, safety, and environmental impacts should also be considered. Most importantly, higher-value cellulose and lignin grades should be produced to improve profitability, and this would require completely different sustainable technologies. Such a transition to producing higher-value cellulose and lignin grades in pulp mills presents numerous R&D challenges, particularly in the development and integration of novel fractionation chemistries (discussed in detail below). While novel technologies hold promise for enhancing efficiency and sustainability, their implementation requires extensive R&D to optimize the conditions to achieve a consistent quality of polymers for specialized applications. This, in turn, requires significant advancements in process control and monitoring, including the development of new characterization techniques and methods. It is also necessary to reduce costs and ensure scalability. Scaling up novel technologies from the lab to commercial production is a lengthy and resource-intensive process, often requiring the construction of pilot and demonstration facilities. This scaling up is further hindered by the lack of existing equipment tailored to these innovative processes, necessitating custom solutions. Moreover, balancing the environmental, health, and safety impacts of new raw materials with their economic viability requires a holistic approach to life-cycle assessment, which can be time-consuming and technically demanding. These R&D efforts are compounded by the need to navigate evolving regulatory frameworks, which often lack clarity regarding new technologies. Overall, the R&D landscape for adopting sustainable innovations in the pulp and paper industry is fraught with technical, economic, and regulatory complexities, demanding sustained investment and interdisciplinary collaboration. Collectively, these challenges contribute to slow and complex commercialization pathways, mirroring the protracted timelines of the legacy technologies they aim to replace.

**Lignin-first approach:** One such alternative to conventional kraft pulping, which focuses on cellulose, is lignin-first biorefining (e.g., BALI™, Borregaard) [149]. Although reductive catalytic approaches have been investigated since the 1940s to produce value-added aromatic chemicals and fuels from lignocellulosic feedstocks [150], the advent of protection-group chemistry to stabilize reaction intermediates has boosted the lignin-first approach since 2015 [151,152,153]. Among its disadvantages are the 2× to 4× higher GHG emissions associated with reductive catalytic fractionation and the potential loss of polysaccharides [154]. Therefore, researchers across the world are collaborating to create guidelines for lignin-first biorefining, including reactor design, mass transfer, and co-product recovery [155,156], to enhance the overall biopolymer yield and quality.

**Hemicellulose-first approach:** Hemicelluloses are easily degraded and are often the sacrificial lambs in lignin-first or cellulose-first biorefining approaches. Hence, many have advocated for integrated biorefining of all biopolymers in kraft pulp mills since 2006 [157], starting with the pre-extraction of hemicelluloses to maximize feedstock utilization efficiency [158]. The GreenBox^+®^ technology, implemented by Cascades (Cabano, QC, Canada) in 2016, is the only known commercial example of hemicellulose hydrolysis and pre-extraction via thermomechanical means [159]. Even minor OPEX (energy, feedstock) increases could not be sustained by the conventional pulp mills [160] and this approach did not materialize on a global scale.

**Organosolv fractionation:** This method simultaneously fractionates lignin and cellulose from a variety of biomasses. First patented in the 1970s [161], it uses a binary or ternary solvent mixture, usually composed of alcohol (ethanol, methanol, butanol) or acid (acetic/formic acid) and water, and sometimes acid catalysts (sulfuric acid) and biphasic additives (methyl isobutyl ketone), to pulp lignocellulosic biomass [162]. Despite significant commercialization challenges [163,164,165], a few chemical and energy companies have set up pilot plants in Germany (Fraunhofer CBP, Leuna) [166], Finland (Fortum, Espoo) [167], Switzerland (Bloom Biorenewables, Marly) [168], and Spain (Fraction project, Madrid) [169]. However, like kraft pulping, the organosolv process produces lower- Mw lignin and completely depolymerized and degraded hemicellulose (Table 2).

**Extrusion-based fractionation:** Another groundbreaking technology for simultaneously fractionating lignin and cellulose involves a patented extrusion method developed by Sweetwater Energy Inc. in 2016 [170], which significantly improved the lignin’s purity (≥92%) and molecular weight (M_w_ = 7500–8280 g/mol) [171]. This technology has been adopted by a couple of European companies, namely Lignovations GmbH (Klosterneuburg, Austria) and Fibenol OÜ (Tallinn, Estonia).

**Table 2:** Examples of source, extraction conditions, and chemical properties of alternative pulping products.

Biomass Source	Pulping Conditions	*M*_w_ of lignin (Da) ^a^	Ref.
***Lignin-first Biorefining***			
Pine	235 °C, 53% w/w methanol, 180 min, Pd/C catalyst	150–550	[172]
***Hemicellulose-first Biorefining***			
Eucalyptus	180 °C, 5% solid loading, 40 min, liquid hot water soluble	560	[173]
Sugar maple	160 °C, 25% solid loading, 45 min, liquid hot water insoluble	2946	[174]
***Organosolv Pulping***			
Birch, Spruce	183 °C, 50–60% *v*/*v* ethanol, 60 min	4200–4600	[175]
*Pinus radiata*	170–200 °C, 40–60 wt.% ethanol, 50–100 min	1650–10,700	[176]
Beech, birch	Acetosolv Fabiola™ process, Fraunhofer CBP	~3500	[177]
Norway spruce	195 °C, 50 wt.% acetone, 1 wt.% H_2_SO_4_, 80–30 min	3300	[178]
***Ionosolv Process***			
*Miscanthus*	Finely ground biomass, 10–20 wt.% loadings, [TEA][HSO_4_] IL/water, T = 120 °C	4620–4900	[179]
*Miscanthus*	Medium-size ground biomass, 10–20 wt.% loadings, [TEA][HSO_4_] IL/water, T = 120 °C	5720–5760	[179]
*Miscanthus*	Coarse biomass, 10–20 wt.% loadings, [TEA][HSO_4_] IL/water, T = 120 °C	5600–6500	[179]
Rice husk	1.5% loading, [C_3_SO_3_Hmim]Cl, T = 100 °C	13,800	[180]
*Miscanthus*	[DMBA][HSO_4_], 80 wt.% in water, precipitated with 0.5 and 1 equiv. water ^b^, 90% total yield	18,200–39,700	[181]
*Miscanthus*	[DMBA][HSO_4_][H_2_SO_4_]0.3 ^c^, 80 wt.% in water, precipitated with 0.25 and 0.5 equiv. water ^b^, 88% total yield	151,800–207,000	[181]
Willow	[DMBA][HSO_4_], 80 wt.% in water, precipitated with 1 and 1.5 equiv. water ^b^, 86% total yield	4400–14,400	[181]
Pine	[DMBA][HSO_4_], 80 wt.% in water, precipitated with 0.5 and 1 equiv. water ^b^, 91% total yield	20,900–75,200	[181]
***Ionosolv/Organosolv Hybrid***			
*Miscanthus*, pine	10–50 wt.% loadings, [TEA][HSO_4_]/Ethanol (80%), T = 120 °C ^d^	~7700	[182]
*Miscanthus*, pine	10–50 wt.% loadings, [TEA][HSO_4_]/Butanol (80%), T = 120 °C ^d^	~7000	[182]
***Eutectic mixtures e-based fractionation***			
*Poplar*	Choline chloride/Lactic acid (1:9 molar ratio) ^e^, wood chips cooked at T = 120 °C	11,374	[183]
*Poplar*	Choline chloride/Lactic acid (1:9 molar ratio) ^e^, wood chips cooked at T = 150 °C	5044	[183]

^a^ M_W_ stands for weight-average molecular weight; ^b^ relative to original IL weight in the solvent; ^c^ Synthesized with acid: base molar ratio 1.03:1, i.e., the composition has 0.3 equiv. of extra acid; ^d^ Best-determined conditions; ^e^ While these are claimed to be DES in the referenced literature, these are simple eutectic mixtures. **Abbreviations:** [TEA][HSO_4_], triethylammonium hydrogen sulfate; [DMBA][HSO_4_], *N*,*N*-dimethylbutylammonium hydrogen sulfate; [C_3_SO_3_Hmim]Cl, 1-methyl-3-(3-sulfopropyl)-imidazolium chloride; *v*/*v*—volume/volume.

**IonoSolv fractionation:** The Ionosolv process [184], developed in 2010 by Imperial College London (IonoSolv, Imperial Innovations, London, UK), is a process in which an ionic liquid (IL) is used as a solvent for biomass. The first IL used for biomass delignification in the IonoSolv process was 1-butyl-1-methylimidazolium methylsulfate ([C_4_C_1_im][C_1_SO_4_]) [185], but later it was replaced with protic [HSO_4_]-based ILs, e.g., triethylammonium hydrogen sulfate ([TEA][HSO_4_]), and N,N-dimethyl-N-butylammonium hydrogen sulfate ([DMBA][HSO_4_]) [186,187]. Protic ionic liquids (PILs) are less expensive than conventional ILs [188] because they are synthesized by a simple proton transfer reaction between an acid and a base [189]. Some of them have been shown to quantitatively delignify lignocellulosic feedstocks at a rate of 53 to 67% [190,191]. PILs can also dissolve pure cellulose by up to 25 wt.% (although this depends on MW of cellulose) [189] and it has been shown that they are capable of recovery and reuse, although this depends on the particular PIL used [181]. IL-based fractionation technology is amenable to a wide variety of feedstock such as miscanthus [192,193], sugarcane bagasse [194], pine [195], and willow [196]. Like organosolv, lignin, and hemicellulose can be extracted from the biomass whereas the crystalline cellulose-rich pulp remains. ILs are usually used in combination with water, which plays an important role in reducing IL viscosity, assisting in lignin removal, and acting as an anti-solvent for cellulose [197].

The most important part of the IonoSolv approach is that the properties of the natural polymers, including M_W_, can be tuned by optimizing the residence time, temperature, pH, and re-precipitation techniques [198,199]. Lignin fractions isolated with minimal water volumes (Table 2) were shown to have the highest MW, a low polydispersity, and a high thermal stability. Such fractional precipitation represents a novel lignin isolation technique that enables a high degree of control over the lignin’s properties, allowing for a wide range of options for specific applications. While the IonoSolv approach has been demonstrated in numerous laboratories, misconceptions about the high cost of all ILs have held back their development.

**Deep eutectic solvents (DESs)-based pulping:** The concept of deep eutectic solvents (DESs) was introduced by Abbott in 2003 [200], who demonstrated the first eutectic mixture composed of ([Cho]Cl) and urea, with a melting point of 12 °C. In his early works, Abbott defined deep eutectic solvent (DES) systems as mixtures formed between quaternary ammonium salts and carboxylic acids. Today, the definition of DESs has expanded to include mixtures of Lewis or Brønsted acidic hydrogen bond donors (HBDs) and basic hydrogen bond acceptors (HBAs) [201], encompassing a wide range of complexes. HBAs typically include quaternary ammonium salts (especially choline chloride, which shows significant melting point depression), phosphonium, and imidazolium salts, while HBDs can be hydrated and non-hydrated metal halides, amides, alcohols (including polyols), organic acids, and carbohydrates [202]. Unlike ILs, whose behavior is dominated by ionic interactions, DESs primarily exhibit HBD–HBA interactions. However, not all component ratios in these mixtures form true DESs, as some may simply remain mixtures, which can be confirmed by constructing a phase diagram.

Alvarez-Vasco et al. evaluated the delignification of hardwood (poplar) and softwood (Douglas fir) using DESs, specifically [Cho]Cl and lactic acid (LA) [203]. Their results demonstrated that the DES treatment removed 79% of lignin from poplar and 58% from Douglas fir under the same conditions. Similarly, Chang and Li compared the delignification of Eucalyptus and rice straw using DESs ([Cho]Cl and LA), suggesting that DESs’ efficacy may vary by species and emphasizing the need for further research to understand the mechanisms behind DES-mediated delignification [204]. Several other studies have explored different ratios between weakly acidic organic acids (such as LA, oxalic acid, formic acid, and glycine) and [Cho]Cl to delignify various lignocellulosic biomasses [205,206,207,208].

DESs have several advantages over traditional solvents, such as being easy to prepare. Several groups have attempted to screen various eutectic solvent systems to determine the most effective one for delignifying biomass. Patented in 2013 (Confederation of European Paper Industries, CEPI aisbl, Brussels, Belgium), DESs were successfully used to fractionate cellulose and 98% of lignin from woodchips [209]. CEPI launched the Provides initiative in 2015 [210]. The Provides project aimed to develop a sustainable, techno-economically feasible pulping technology for wood and agro-based lignocellulose materials using DESs capable of dissolving and fractionating lignin, hemicellulose, and cellulose at low temperatures and atmospheric pressure, supporting the European pulp and paper industry’s transition to a low-carbon bioeconomy. The project involved selecting effective DES families, developing processing techniques, and assessing environmental, economic, and energy impacts. The premise of the initiative was the use of pure lignin isolated in the DES pulping process to replace aromatics in the chemical industry (instead of being used for heat or electricity), with much higher energy savings of over 160,000 GWh annually and a 10% reduction in CO_2_ emissions. Taking into account the simultaneous production of an equal tonnage of chemicals, the total energy use and CO_2_ emissions of the combined system were estimated to be 90% lower than those with the separate systems of pulp and fossil chemicals production.

Its successor, the Prides project was launched in 2019 to continue building techno-economically feasible, DES-based pulping technologies [211]. Under the acidic environment of lactic acid and lignin *β*-*O*-4 bond cleavage induced by chloride ions, this DES can delignify lignocellulosic feedstocks. Earlier studies reported lignin co-products with very low MW (~1800 Da), but as shown in Table 2, recent optimizations have improved the lignin yield and quality (high MW, purity) (albeit not at all to the extent achievable with the IonoSolv process) [212].

Although DES pulping is another promising and sustainable alternative to conventional methods, the effectiveness of delignification depends on factors such as the complexity of the lignocellulosic biomass, the specific DES used, and the delignification conditions. While DESs have been increasingly used for biomass fractionation due to their high selectivity in delignification, which preserves cellulose and hemicellulose, these systems may have a limited solubility for certain biomass components and may require higher processing temperatures compared to other methods. The use of DESs in biomass delignification is still evolving, and further research is needed to optimize these systems, assess their environmental impact, and determine their potential as alternatives to traditional solvents.

## 7. Sustainability Is Hard, but When Do the Hard Decisions Get Made?

In the bio-renewables sector, many US manufacturers seem to adhere to an outdated mindset toward new product development which overemphasizes short-term cost-based strategies. Such efforts focus on a narrow field of vision and limit the number and type of products potentially developed. In addition to the current “top-down” approaches, which pursue the production of goods from a specific biopolymer (e.g., lignin or cellulose) and dedicate all efforts towards maximizing its output, a “bottom-up” approach will investigate what products can be manufactured from a particular biomass based on its properties (e.g., strength, crystallinity, absorbency) and thereby reduce energy and other inputs. This ‘holistic integrative approach’ towards the manufacturing of products from lignocellulose will not only allow for a to switch to biodegradable resources but will also result in product expansion and diversification. Other advantages would include the ability to utilize biomass fully (without creating ‘waste out of waste’), develop products faster, incorporate the best technologies into different products, and expend fewer resources.

The problem here is the misplaced reliance on a 200-year-old chemical pulping technology for a new sustainable future among scientists, engineers, academics, industry, and government. While innovations in wood pulping, as discussed in Section 6.3, show promising advances in sustainability and efficiency, there are limitations to their current scalability. These innovations, such as alternative biomass processing techniques, are often not yet at a scale capable of meeting the growing demand for paper products, especially in the packaging sector. The rapid increase in global packaging consumption requires large-scale, cost-effective production, which current pulping innovations may not yet support. For these advancements to have a meaningful impact, further scaling and optimization of these technologies are necessary to meet industrial demands.

Making excuses to deny investment or research funding in new techniques to source natural polymers, by labeling them as being too expensive or ill-suited for existing formulae, is a recipe for completely blocking innovation. One only has to look (objectively) at the current environmental trends and data to understand what will happen if we do not innovate and rely on ‘business as usual’.

## Figures and Tables

**Table 1:** Effect of kraft pulping on lignin isolation from different lignocellulosic feedstocks.

Source	Pulping Conditions	MW After Pulping (Da)	Ref.
Anatolian black pine	170 °C, 18% AA ^a^, 30% sulfidity, 180 min	6395	[101]
Poplar	170 °C, 16% AA, 26% sulfidity, 150 min	4061	[101]
Pine and Spruce (LignoForce™)	170 °C, 17.5% EA ^b^, 120 min	4836	[102]
*Acacia* spp. and *Quercus* spp.	160–165 °C, 21–23% EA, 23% sulfidity	3041	[95]
Loblolly pine	170 °C, 13.4% AA, 30 min	3350–4400	[97]

^a^ AA stands for active alkali charge which measures total [OH]^−^ and [HS]^−^ ions; ^b^ EA stands for effective alkali charge which indicates only the [OH]^−^ ion concentration. Please note: The Mw (weight-average molecular weight) results are limited by the measurement technique, i.e., gel permeation chromatography, and the reference standard (i.e., polystyrene).
